# Diagnostic performance of individual characteristics and anthropometric measurements in detecting elevated serum alanine aminotransferase among children and adolescents

**DOI:** 10.1186/s12887-020-02033-9

**Published:** 2020-03-20

**Authors:** Yu-Lan Ou, Yue-Rong Lai, Chao-Nan Jiang, Jing Zhang, Zan Ding

**Affiliations:** 1 Department of Gynecology, Sun Yat-sen University Cancer Center, Guangzhou, 510060 Guangdong China; 2 The Institute of Metabolic Diseases, Baoan Central Hospital of Shenzhen, The Fifth Affiliated Hospital of Shenzhen University, Shenzhen, Guangdong 518102 P.R. China

**Keywords:** Adolescent, Alanine aminotransferase, Anthropometric measurement, Body mass index, BMI-*z*

## Abstract

**Background:**

Screening for elevated serum alanine aminotransferase (ALAT) can help identifying individuals at the risks of chronic and metabolic diseases, but blood collection is invasive and cannot be widely used for investigations. Considered as simple and inexpensive screening indices, individual characteristics and anthropometric measurements can be measured in a large crowd and may be important surrogate markers for ALAT levels. This study aimed to examine the diagnostic performance of individual characteristics and anthropometric parameters as predictive factors for discerning an elevated ALAT activity among Shenzhen children and adolescents.

**Methods:**

A school-based screening study was performed from 9 high schools in Shenzhen during February 2017 and June 2018. Receiver operating characteristic curve was used to examine the diagnostic performance of each variable for detecting elevated ALAT.

**Results:**

Altogether 7271 students aged 9–17 years were involved. The proportion of elevated ALAT greatly increased with increasing classification of BMI-*z*. By the sex-specific cut-offs for elevated ALAT (30 U/L boys; 19 U/L girls), BMI showed the highest area under the curve of 0.789 (95% CI 0.765–0.812) and followed by weight (0.779 [0.755–0.802]), BMI-*z* (0.747 [0.722–0.772]), height (0.622 [0.597–0.647]), and age (0.608 [0.584–0.632]), while height-*z* was not capable. With the cut-off of 67.8 kg for weight and 22.6 kg/m^2^ for BMI, the accuracy to identify elevated ALAT was 87.1% for weight and 82.9% for BMI.

**Conclusions:**

The presence of elevated ALAT was more common in overweight or obese children and adolescents. BMI and weight had the superiority of detecting elevated ALAT, followed by BMI-*z*, height, and age.

## Background

The liver enzyme of serum alanine aminotransferase (ALAT or ALT) could be used clinically as a screening tool for the detection of probable nonalcoholic fatty liver disease (NAFLD), potential liver dysfunction, viral hepatitis infection, hepatocellular damage, and infectious mononucleosis [1–7]. An elevation of serum ALAT concentration is a key feature of NAFLD, and liver biopsy is the gold standard to confirm the diagnosis of NAFLD, characterized by pathological changes to liver structure and function such as fat accumulation, hepatocyte dysfunction, and fibrosis [7]. In addition, the elevation of ALAT has been recorded by published evidence to be strongly linked to, or to predict, the developing of type 2 diabetes mellitus, insulin resistance, and cardiovascular diseases (e.g. atherogenesis, congestive heart failure and coronary heart disease) [5, 6, 8–14].

Screening for the concentration of serum ALAT and verifying an elevated ALAT activity could assist in determining individuals at risk for the above chronic conditions and reducing the possibility of future liver diseases [15, 16]. However, the measurement of ALAT levels requires blood collection, which is invasive and could be impractical in large-scale epidemiological investigations because of subject burden and cost. In contrast, individual characteristic (e.g. age and gender) and measurements of anthropometric indices (e.g. height, weight, body mass index [BMI], and waist circumference [WC]) are relatively simple, cheap, quick, and non-invasive, and therefore can be applied to a great number of people and easily conducted during common health examinations, especially for non-adults [15, 17, 18]. If these indices showed a close correlation with serum ALAT levels or had sufficient ability to detect an ALAT elevation, they could be a useful surrogate for ALAT levels.

Two multi-center, population-based epidemiological researches conducted in the mainland of China have consistently demonstrated that the higher values of BMI, hip circumference, WC, waist-to-hip ratio (WHpR), and waist-to-height ratio (WHtR) were able to predict an elevated ALAT activity [17, 19]. Recently, the nontraditional index of BMI z-score has also been put forward as another important anthropometric indicator of ALAT elevation [7, 20]. However, whether the potential of the *z*-score of height, a novel body index, for predicting elevated ALAT was comparable or superior to the abovementioned parameters was still unclear up to now. In addition, although most previous studies on the relations of anthropometric measurements and individual characteristics with ALAT elevation were conducted among adults [17, 21–23], few investigations focused on the performance predication of those predictors for diagnosing elevated ALAT among children and adolescents [15, 20, 24, 25].

Based on a school-based screening, the current study mainly aimed to examine the diagnostic performance of individual characteristics (i.e. age and gender) and several anthropometric parameters (i.e. height, weight, BMI, BMI-*z*, and height-*z*) as predictive factors of an elevated ALAT activity in Shenzhen children and adolescents, and to determine the optimal cut-off points for these parameters that would identify a person with an elevation of serum ALAT level. Assessing individual indicators is crucial for early detection and prevention of an activity of elevated ALAT or the subsequent development of NAFLD among children and adolescents.

## Methods

### Study setting and population

Shenzhen is one of the 4 first-tier cities in mainland China and a major financial centre and the worldly largest manufacturing base [26]. The population and economy have grown rapidly, with a total gross domestic product of 255 billion USD in 2015 [27].

### Data collections and measures

Involved freshmen from 9 junior and senior high schools of Bao’an District, a cross-sectional study organized with the assistance of the Bao’an Government of Shenzhen was officially carried out [28]. Data collection activities were completed during February 2017 and June 2018. This school-based study was approved by the Institutional Review Board of Baoan Central Hospital of Shenzhen, and written informed consents for each student and their parents were obtained.

Individual characteristic data such as gender and date of birth were taken from each participant, and anthropometric parameters of weight and standing-height were respectively measured to the nearest 0.1 kg and 0.5 cm by trained physicians using a standard weighing machine. At each high school, blood samples were drawn in the morning after participants fasting for at least 10 h by trained nurses. ALAT was measured with the fully automatic biochemical analyzer (Model AU5821, Tokyo, Japan). Finally, a total of 7271 students who had complete anthropometric and clinical data participated.

For each participant, age with 1 decimal was transformed based on the date of birth and BMI (kg/m^2^) was calculated in weight divided by the square of height. Based on an international norm from the World Health Organization growth reference (i.e. the 2007 WHO reference) for school-aged children and adolescents aged 5–19 years, anthropometric indicators of z-score values of height-for-age (height-*z*) and BMI-for-age (BMI-*z*) for both boys and girls were calculated by the R macro, provided on the WHO website (*https://www.who.int/growthref/en/*) [29, 30]. Moreover, according to the z-score of BMI-for-age, we defined BMI-*z* > 2SD as obesity, 1SD < BMI-*z* ≤ 2SD as overweight, −2SD ≤ BMI-*z* ≤ 1SD as normal-weight, and BMI-*z* < −2SD as underweight; where SD was standard deviation of the BMI z-scores.

### Definition of elevated serum ALAT levels

The thresholds of elevated ALAT needed to be determined consistent with previously reported levels, as there was no consensus on what level constituted an elevated serum ALAT concentration for children and adolescents. For the most commonly use of definitions among children and adolescents, an activity of elevated serum ALAT was defined as > 30 U/L for boys and > 19 U/L for girls (diagnostic criterion I) [5, 6, 11, 31]. Main analysis was undertaken using these sex-specific cut-offs, and a secondary analysis for the sake of comparison was also reported, defining with higher thresholds (> 40 U/L for both boys and girls; diagnostic criterion II) that have been proposed in previous observational literatures [10, 16, 32] to explore whether higher thresholds led to the same or different results.

### Statistical analyses

The normal distribution of each continuous measurement was determined by the one-sample Kolmogorov-Smirnov test. The non-normally distributed measurements were described as median with inter-quartile range (25th percentile−75th percentile; IQR); significance for differences between elevated and normal serum ALAT was evaluated by the Mann–Whitney U test. Stratified by the classification of BMI *z*-score, the crude prevalence along with 95% confidence interval (CI) of elevated ALAT was quantitatively estimated, and the trend of the proportion of elevated ALAT based on BMI-*z* was carried out with chi-square test. The Spearman’s rank correlation analyses among the skewed parameters were performed.

Extensively used in clinical epidemiology for evaluation of diagnostic ability of biomarkers (e.g. serum markers) or a diagnostic test with dichotomous outcome (i.e. positive or negative result) in classification of the diseased from healthy population, the receiver operating characteristic (ROC) curve, defined as a plot of the sensitivity of a test as y-axis versus 1-specificity as x-axis, is an effective method and graphical technique to describe the accuracy of a prediction model or diagnostic test [33, 34]. Sensitivity and specificity, the basic measures of accuracy of a diagnostic test, vary with different cut-off thresholds, and sensitivity is inversely related to specificity [18, 34]. Positive predictive value (PPV) is defined as the probability of disease for positive test results, and negative predictive value (NPV) is defined as the probability of being healthy for negative test results [33].

In the current study, we utilized the ROC curve analysis to find out the optimal cut-off points and to examine the diagnostic performance of each measurement as indicators of ALAT elevation. An anthropometric parameter value with the highest Youden index was chosen as the optimal cut-off point [18]. The observed agreement and the area under the curve (AUC) were also determined. Statistical analyses were processed in R 3.5.1 (*http://www.R-project.org*) and SPSS for Windows 16.0, with two-tailed *P*-value < 0.05 considered statistically significant.

## Results

### Characteristics of participants

In the current investigation, a total of 7271 students aged 9–17 years were recruited for the operative statistical analysis, involving 4014 (55.2%) boys. Characteristics of study subjects with and without elevated ALAT activity are listed in Table 1. The indices of age, weight, height, BMI, height-*z*, and BMI-*z* were non-normal distribution (all *P* < 0.001). Overall, the median (IQR) level was 14.7 (12.4–15.7) years for age, 53.0 (46.7–61.0) kg for weight, 1.65 (1.59–1.72) m for height, 19.31 (17.63–21.66) kg/m^2^ for BMI, 0.59 (− 0.18 to 1.41) for height-*z*, and − 0.01 (− 0.77 to 0.83) for BMI-*z*. Each anthropometric index but not height-*z* was significantly greater among students with an elevated ALAT activity than those with a normal ALAT level, regardless of the use of definitions (most *P* < 0.001).

**Table 1:** Baseline descriptive statistics for individual measurements stratified by gender among Shenzhen children and adolescents (aged 9–17 years), separately based on the diagnostic criteria I and II for elevated ALAT

Group	Total students	Criterion I (> 30 U/L for boys and > 19 U/L for girls)			Criterion II (> 40 U/L for boys and girls)		
		Normal ALAT	Elevated ALAT	*P*-value^*^	Normal ALAT	Elevated ALAT	*P*-value^*^
Overall (*n* = 7271)							
Age, years	14.7 (12.4–15.7)	14.7 (12.3–15.7)	15.3 (13.8–16.1)	< 0.001	14.7 (12.3–15.7)	15.3 (14.3–16.1)	< 0.001
Height, m	1.65 (1.59–1.72)	1.64 (1.59–1.71)	1.69 (1.63–1.75)	< 0.001	1.65 (1.59–1.72)	1.73 (1.65–1.78)	< 0.001
Weight, kg	53.0 (46.7–61.0)	52.2 (46.2–59.8)	70.0 (56.1–83.6)	< 0.001	52.6 (46.5–60.2)	77.7 (63.1–90.1)	< 0.001
BMI, kg/m^2^	19.31 (17.63–21.66)	19.15 (17.55–21.23)	24.51 (20.38–27.95)	< 0.001	19.25 (17.59–21.45)	26.39 (22.40–30.22)	< 0.001
Height-*z*	0.59 (− 0.18–1.41)	0.58 (− 0.18–1.41)	0.71 (− 0.12–1.47)	0.104	0.59 (− 0.18–1.41)	0.65 (− 0.13–1.48)	0.526
BMI-*z*	− 0.01 (− 0.77–0.83)	−0.06 (− 0.81–0.74)	1.40 (0.16–2.27)	< 0.001	−0.04 (− 0.79–0.79)	1.89 (0.85–2.54)	< 0.001
Boys (*n* = 4014)							
Age, years	14.7 (12.4–15.7)	14.7 (12.3–15.7)	15.4 (14.4–16.1)	< 0.001	14.7 (12.4–15.7)	15.4 (14.6–16.2)	< 0.001
Height, m	1.69 (1.61–1.75)	1.69 (1.61–1.75)	1.74 (1.68–1.78)	< 0.001	1.69 (1.61–1.75)	1.74 (1.68–1.79)	< 0.001
Weight, kg	55.4 (48.1–64.7)	54.7 (47.9–63.0)	79.0 (66.9–88.6)	< 0.001	55.0 (48.0–63.7)	81.4 (69.5–92.4)	< 0.001
BMI, kg/m^2^	19.37 (17.64–22.04)	19.16 (17.55–21.47)	26.42 (22.98–30.06)	< 0.001	19.26 (17.59–21.70)	27.20 (23.92–30.61)	< 0.001
Height-*z*	0.65 (−0.16–1.55)	0.65 (− 0.16–1.55)	0.66 (− 0.16–1.42)	0.086	0.65 (− 0.16–1.55)	0.66 (− 0.12–1.50)	0.857
BMI-*z*	0.12 (− 0.74–1.06)	0.04 (− 0.78–0.90)	1.98 (0.99–2.55)	< 0.001	0.07 (−0.77–0.95)	2.14 (1.27–2.67)	< 0.001
Girls (*n* = 3257)							
Age, years	14.7 (12.3–15.7)	14.7 (12.3–15.7)	15.0 (13.3–16.0)	< 0.001	14.7 (12.3–15.7)	14.6 (13.3–15.4)	0.557
Height, m	1.62 (1.57–1.67)	1.61 (1.57–1.66)	1.64 (1.58–1.71)	< 0.001	1.62 (1.57–1.66)	1.62 (1.58–1.69)	0.270
Weight, kg	50.6 (45.2–57.0)	50.1 (45.0–56.0)	58.5 (50.6–71.1)	< 0.001	50.5 (45.2–56.8)	59.0 (48.7–75.6)	< 0.001
BMI, kg/m^2^	19.26 (17.60–21.26)	19.13 (17.54–20.98)	21.60 (19.05–25.59)	< 0.001	19.23 (17.60–21.19)	22.01 (18.86–27.12)	< 0.001
Height-*z*	0.51 (−0.20–1.32)	0.50 (− 0.20–1.31)	0.82 (− 0.09–1.52)	0.006	0.51 (− 0.20–1.32)	0.40 (− 0.20–1.37)	0.857
BMI-*z*	−0.13 (− 0.82–0.61)	−0.16 (− 0.84–0.54)	0.64 (− 0.39–1.60)	< 0.001	−0.14 (− 0.82–0.60)	0.73 (− 0.53–1.80)	< 0.001

Data are expressed as median (interquartile range [IQR]) for the measurements with skewed distributions

^*^ Comparing elevated ALAT to normal ALAT by the Mann–Whitney U test

### Crude prevalence of elevated ALAT by the BMI-*z* classification

On the basis of ALAT activity thresholds of diagnostic criterion I, an elevated ALAT activity was present in 2.48% (6/242) of underweight, 3.79% (208/5493) of normal-weight, 12.58% (137/1089) of overweight, and 36.91% (165/447) of obese children and adolescents; based on the criterion II, the proportion of elevated ALAT was 0.83% (2/242), 0.89% (49/5493), 4.96% (54/1089), and 20.58% (92/447) for the corresponding subgroups (Table S1). Regardless of the use of diagnostic criteria, the abnormality prevalence rate of ALAT greatly increased with increasing classification of BMI z-score and peaked in the obesity group for all participants, boys, and girls (all *P*_*trend*_ < 0.001). Overweight or obese students were much more likely to obtain increased ALAT concentrations than normal-weight or underweight students.

### Univariate ROC curve analyses

**Fig. 1:**
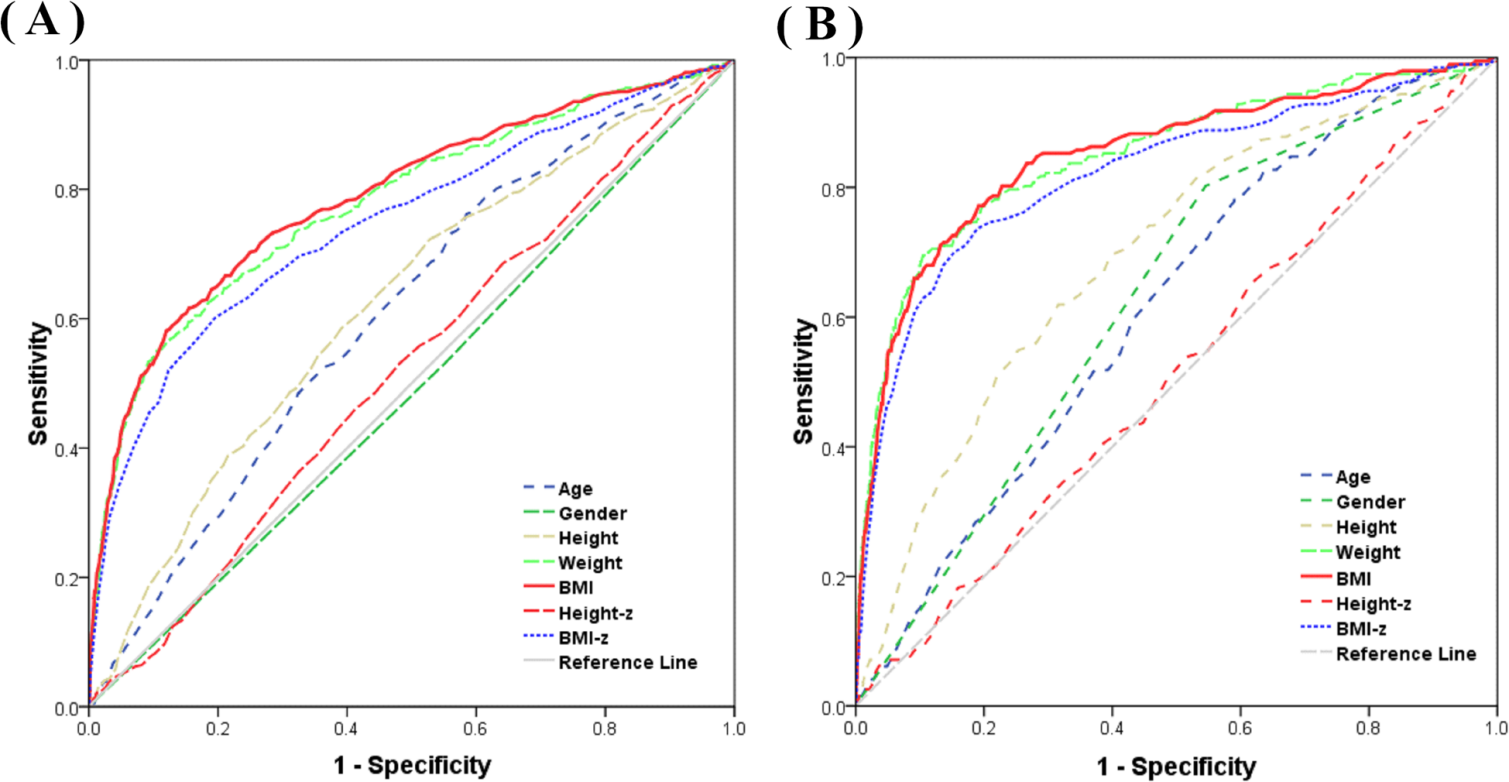
ROC curve of individual characteristics and anthropometric measurements as indicators of elevated serum ALAT among the Shenzhen children and adolescents, separately based on the diagnostic criteria I (panel **a**) and II (panel **b**)

Figure 1 illustrates the diagnostic performance of individual indices for identifying elevated ALAT by ROC curves, and Table 2 presents the information about the AUCs of the curves among Shenzhen children and adolescents. The anthropometric parameter of BMI showed the highest AUC (95% CI) for elevated ALAT (0.789 [0.765–0.812] by criterion I; 0.850 [0.818–0.882] by criterion II), and followed by weight (0.779 [0.755–0.802]; 0.850 [0.817–0.882]) and the z-score of BMI (0.747 [0.722–0.772]; 0.822 [0.787–0.858]). Compared to the above 3 obesity indices, age and height had a poorer diagnostic efficiency in detecting abnormal ALAT. The discriminatory power of height z-score in the prediction of elevated ALAT was null (both *P* > 0.1), and gender also had no ability to evaluate elevated ALAT by the diagnostic criterion I (*P =* 0.435). In the stratification analyses by gender, similar outcomes were provided in Table S2.

**Table 2:** The AUC (95% CI) of the ROC curve to judge the discrimination ability of various individual measurements for detecting elevated ALAT (separately by the criteria I and II) in univariate analyses

Variable	Criterion I (> 30 U/L for boys and > 19 U/L for girls)				Criterion II (> 40 U/L for boys and girls)			
	AUC	95% CI	SE	P^*^	AUC	95% CI	SE	P^*^
Age	0.608	0.584–0.632	0.012	< 0.001	0.613	0.577–0.649	0.018	< 0.001
Gender	0.490	0.464–0.516	0.013	0.435	0.628	0.593–0.664	0.018	< 0.001
Height	0.622	0.597–0.647	0.013	< 0.001	0.695	0.658–0.732	0.019	< 0.001
**Weight**	**0.779**	**0.755–0.802**	**0.012**	**< 0.001**	**0.850**	**0.817–0.882**	**0.016**	**< 0.001**
**BMI**	**0.789**	**0.765–0.812**	**0.012**	**< 0.001**	**0.850**	**0.818–0.882**	**0.016**	**< 0.001**
Height-*z*	0.522	0.496–0.547	0.013	0.102	0.514	0.473–0.554	0.021	0.517
**BMI-*z***	**0.747**	**0.722–0.772**	**0.013**	**< 0.001**	**0.822**	**0.787–0.858**	**0.018**	**< 0.001**

*AUC* Area under curve, *ALAT* Alanine aminotransferase, *CI* Confidence interval, *ROC* Receiver operating characteristic, *SE* Standard error

^*^ Null hypothesis: true area = 0.5

Table 3 elucidates the optimal cut-off points and the probability of meaningful and significant parameters in diagnosing abnormal ALAT, based on ROC analyses. The suggested cut-off points of were 67.8 & 68.9 kg for weight and 22.6 & 22.3 kg/m^2^ for BMI, separately by the criteria I and II. Based on the cut-offs, the parameter of weight achieved the highest ability to correctly classify subjects as elevated ALAT, with an accuracy of 87.1% by criterion I and 88.9% by criterion II. However, BMI took control of a larger Youden index (46.1%) than other parameters with criterion I. BMI and weight had an approximate diagnostic performance in diagnosing elevated ALAT.

**Table 3:** The optimal cut-off points and the probability of significant individual parameters for identifying elevated ALAT, separately based on the diagnostic criteria I and II

Parameter	Criterion I (> 30 U/L for boys and > 19 U/L for girls)							Criterion II (> 40 U/L for boys and girls)						
	Cut-off	Accuracy	Sen	Spe	Youden index	PPV	NPV	Cut-off	Accuracy	Sen	Spe	Youden index	PPV	NPV
Age	13.7 years	44.0	76.2^a^	41.5	17.7	9.0	95.8	13.7 years	41.8	78.7^a^	40.8	19.5	3.6	98.6
Height	164.0 cm	49.0	72.3	47.3	19.5	9.5	95.7	169.5 cm	67.3	61.9	67.5	29.4	5.0	98.5
Weight	67.8 kg	87.1^a^	55.0	89.5^a^	44.6	28.6^a^	96.3	68.9 kg	88.9^a^	69.5	89.5^a^	59.0^a^	15.6^a^	99.1
BMI	22.6 kg/m^2^	82.9	61.6	84.5	46.1^a^	23.3	96.6^a^	22.3 kg/m^2^	80.8	77.2	80.9	58.0	10.1	99.2^a^
BMI-*z*	0.95	79.1	60.1	80.5	40.6	19.1	96.4	1.25	84.3	70.1	84.7	54.7	11.3	99.0

The optimal cut-off points were based on the maximum Youden index for each parameter; the probability of parameters for identifying elevated ALAT is expressed as %

*Sen* Sensitivity, *Spe* Specificity, *PPV* Positive predictive value, *NPV* Negative predictive value

^a^ The best performance for each conventional diagnostic test index

### Correlation analyses and combined individual measurements for detecting elevated ALAT in multivariate analysis

Table S3 displays the Spearman’s correlation coefficients between individual indices and ALAT levels. BMI had a very high positive correlation with BMI *z*-score (*r* = 0.891) and weight (*r* = 0.829), suggesting a multicollinearity among each obesity parameter. Weight was also quite strongly and positively correlated with height (*r* = 0.673) and BMI-*z* (*r* = 0.657).

Table 4 shows the capability of combined individual indices to diagnose the presence of elevated ALAT in multivariate models. The combined indices of “age + gender+height + weight” showed the strongest capability to indicate the levels of ALAT, with an AUC (95% CI) of 0.816 (0.795–0.836) for criterion I and 0.858 (0.826–0.891) for criterion II. In particular, a very slight increase of 0.037 (0.816 vs 0.779) for criterion I and 0.008 (0.858 vs 0.850) for criterion II in accuracy was demonstrated, as compared with the univariate weight model. The multivariate combined BMI model also fitted well with an analogous good accuracy (AUC = 0.813 for criterion I and 0.858 for II). Compared to the univariate models of obesity indices (i.e. weight, BMI, or BMI-*z*), the combined models did not markedly improve the prediction of elevated ALAT for children and adolescents.

**Table 4:** The AUC (95% CI) of the ROC curve to express the diagnostic performance of combined individual measurements for detecting elevated ALAT (separately by the diagnostic criteria I and II) in multivariate analyses

Combined measurements	Criterion I (> 30 U/L for boys and > 19 U/L for girls)				Criterion II (> 40 U/L for boys and girls)			
	AUC	95% CI	SE	P^*^	AUC	95% CI	SE	P^*^
Age + gender+height	0.643	0.619–0.668	0.012	< 0.001	0.716	0.679–0.753	0.019	< 0.001
Age + gender+height + weight	0.816	0.795–0.836	0.011	< 0.001	0.858	0.826–0.891	0.016	< 0.001
Age + gender+height + BMI	0.813	0.792–0.834	0.011	< 0.001	0.858	0.826–0.890	0.016	< 0.001
Age + gender+height + BMI-*z*	0.807	0.785–0.829	0.011	< 0.001	0.856	0.823–0.889	0.017	< 0.001

*AUC* Area under curve, *ALAT* Alanine aminotransferase, *CI* Confidence interval, *ROC* Receiver operating characteristic, *SE* Standard error

^*^ Null hypothesis: true area = 0.5

## Discussion

Primary focused on the discriminative accuracy of the indices, individual characteristic and anthropometric data for Shenzhen children and adolescents aged 9–17 years were used to diagnose elevated ALAT, and we confirmed the belief that individual indices were important predictors of abnormal ALAT. An elevated ALAT activity was more common in overweight or obese students than those with a normal or relatively low BMI *z*-score. BMI and weight showed an approximate diagnostic performance for predicting the presence of elevated ALAT, followed by the *z*-score of BMI, height, and age.

Consistent findings that the proportion of elevated ALAT greatly increased with increasing degree of obesity (e.g. the percentile or z-score of BMI) and higher proportion among overweight or obese adolescents than normal-weight ones were shown in previous studies and our research, regardless of the use of diagnostic criteria for an elevated ALAT activity [10, 16, 21, 35]. Based on the criterion II (40 U/L for boys and girls), the percentage of elevated ALAT among Shenzhen children and adolescents of 9–17 years was 0.89% for normal-weight, 4.96% for overweight, and 20.58% for obese participants. According to the same thresholds, elevated ALAT levels were observed in nearly 6.6% of the Mexican youths (9.8% of boys and 3.8% of girls), and 2.7% of normal-BMI, 14.2% of overweight, and 28.9% of the obese children and adolescents, by using the baseline data from 1262 participants of 8–19 years in the Mexican Health Worker Cohort Study [16]. Mexican youths had a slightly higher crude proportion of elevated ALAT than Shenzhen youths, without adjusting for age and gender based on the distribution of the world population. Likewise, using a sample of 1591 youths from the 2008–2009 Korea National Health and Nutrition Examination Survey, another study conducted by Seung Park and cooperators also found a close prevalence of elevated ALAT (> 33 U/L for boys and > 25 U/L for girls) among Korean youths of 12–18 years — 5.9% (95%CI 4.9%–7.2%) for the overall study population, 15.7% (11.3%–21.5%) for overweight adolescents (85th ≤ BMI < 95th percentile), and 34.9 (25.6%–45.4%) for subjects with a BMI ≥ 95th percentile [35]. The odds ratios (ORs) for elevated ALAT also sharply increased with the greater levels of obesity, with an OR (95% CI) of 7.23 (4.33–12.10) for overweight and 23.62 (12.98–42.98) for obese adolescents, comparing to normal-weight adolescents (BMI < 85th percentile) in an unadjusted analysis [35].

Screening for school-students, it is clearly more convenient to collect anthropometric measures than biochemical measures, but the abilities of several individual characteristics and anthropometric indices to correctly predict elevated serum ALAT in children and adolescents are questionable and need to be assessed. Determined by the univariate ROC curves, our results showed that the anthropometric parameter of BMI had the best superiority of discerning the presence of elevated ALAT (AUC = 0.789 for criterion I and 0.850 for II), weight and BMI-*z* displayed the second and third highest detection accuracy (from 0.747 to 0.850), and followed by height, age, and gender (from 0.490 to 0.695). As such, we believed that the single variable of height, age, or gender was not a reliable surrogate measure of an activity of elevated ALAT among Shenzhen students of 9–17 years. Further, age, gender, and height were considered as covariates together with each predictor of obesity indices (i.e. weight, BMI, or BMI-*z*) to predict elevated ALAT in the subsequent multivariate model analyses, although the diagnostic accuracy of the combined models did not markedly improve. On the other hand, the current study firstly investigated the association of height z-score with serum ALAT and estimated the usefulness of height-*z* as a predictive index of elevated ALAT among children and adolescents, showing a null discriminative power of height *z*-score for predicting an elevated ALAT activity.

For BMI, several studies had pointed out that BMI was able to determine the presence of elevated ALAT among adults, although the accuracy was not very high in absolute term — the AUC (95% CI) was 0.658 (0.633–0.683) for men and 0.651 (0.616–0.685) for women in the rural areas of China [17] and 0.64 (0.60–0.68) for Italian general population [22]. Focused on adolescents, a representative study with a sample of 454 youths of 11–17 years from 2 northern Italian cities also found a similar diagnostic performance of dichotomized BMI for elevated ALAT, with an AUC of 0.64 (0.50–0.77) [20]. Compared to the dichotomized BMI model, the Italian adolescents study also indicated a more accurate univariate model of BMI-*z* (AUC = 0.71 [0.59–0.81]), and the predictive ability was increased substantially by considering gender together with BMI-*z* (AUC = 0.80 [0.71–0.89]) [20]. In addition, based on the data from the National Health and Nutrition Examination Survey during 1999 to 2014, an United States study consisted of 5019 adolescents of 12–19 years suggested a significant correlation between BMI z-score and serum ALAT levels (*r* = 0.29, *P* < 0.0001) [7]. Significant positive associations of ALAT with obesity indices (e.g. BMI, body fat percentage, truncal fat mass, total fat mass, WC, and WHtR) were further confirmed in Korean male adolescents [24].

Several potential limitations of our research should be recognized. First, participants were only from a single city of China, and it might be therefore difficult to generalize these findings to other populations. Second, our cross-sectional study omitted to measure some important individual anthropometric parameters — hip circumference, WC, neck circumference, and the subsequent indicators of WHpR, WHtR, and A Body Shape Index, which might achieve a better diagnostic performance and to be more sensitive predictive indicates for diagnosing an elevated ALAT activity among the indigenous adolescents. For predicting the ALAT levels, WHtR and to some extent BMI were congruously shown to be the best body indices in some Asians, as compares to WC, hip circumference, WHpR, and the useless index of A Body Shape Index [15, 17, 19]. Third, our study did not take into account some potential confounders of ALAT elevation for non-adults such as physical exercise, drugs, and dietary choline deficiency [1]. Another limitation was our inability to consider the potential confounders of ethyl alcohol intake and hepatitis B virus (HBV) and C virus (HCV) infections, which were well-known risk factors for increases in ALAT [7, 15, 20, 22]. However, alcohol consumption and HBV and HCV infections were less prevalent in school-students of China [36, 37], and we hypothesized that the obesity indices could be even more important predictors of elevated ALAT among the children and adolescents of Shenzhen, and the above potential confounders were not likely to have a substantial impact on current results.

## Conclusions

An elevated ALAT activity was more frequent in overweight or obese students than normal-weight subjects. Anthropometric obesity indices of BMI, weight, and BMI-*z* achieved a very high diagnostic performance for predicting elevated ALAT among Shenzhen children and adolescents and followed by height and age, while height-*z* was not capable.

## Supplementary information

**Additional file 1: Table S1.** Crude prevalence of elevated ALAT stratified by the classification of BMI *z*-score among children and adolescents of Shenzhen, separately based on the diagnostic criteria I and II. **Table S2.** The AUC (95% CI) of the ROC curve stratified by gender in univariate analyses, separately based on the diagnostic criteria I and II for elevated ALAT. **Table S3.** Spearman’s rank correlation coefficients between individual measurements and ALAT.

## Data Availability

Readers can get the datasets and materials of the current study by contacting the corresponding author of Zan Ding (dingzan_1990@163.com) for a reasonable request.

## Abbreviations

ALAT: Alanine aminotransferase
AUC: Area under the curve
BMI: Body mass index
CI: Confidence interval
HBV: Hepatitis B virus
HCV: Hepatitis C virus
NPV: Negative predictive value
NAFLD: Nonalcoholic fatty liver disease
PPV: Positive predictive value
ROC: Receiver operating characteristic
SD: Standard deviation
WC: Waist circumference
WHpR: Waist-to-hip ratio
WHtR: Waist-to-height ratio
