# Supplementary material for: Diagnostic performance of individual characteristics and anthropometric measurements in detecting elevated serum alanine aminotransferase among children and adolescents
Source: BMC Pediatr. 2020 Mar 20;20:131. doi: 10.1186/s12887-020-02033-9 (PMC7082986; doi:10.1186/s12887-020-02033-9)
Supplement: Supplementary file 1 — Additional file 1: Table S1. Crude prevalence of elevated ALAT stratified by the classification of BMI z-score among children and adolescents of Shenzhen, separately based on the diagnostic criteria I and II. Table S2. The AUC (95% CI) of the ROC curve stratified by gender in univariate analyses, separately based on the diagnostic criteria I and II for elevated ALAT. Table S3. Spearman’s rank correlation coefficients between individual measurements and ALAT. [file 12887_2020_2033_MOESM1_ESM.doc]

**Table S1.** Crude prevalence of elevated ALAT stratified by the classification of BMI *z*-score among children and adolescents of Shenzhen, separately based on the diagnostic criteria I and II.

| Classification  of BMI-z | Criterion I (> 30 U/L for boys and > 19 U/L for girls) | | |  | Criterion II (> 40 U/L for boys and girls) | | |
| --- | --- | --- | --- | --- | --- | --- | --- |
|  | Overall | Boys | Girls |  | Overall | Boys | Girls |
| Overall | 7.10 (6.51－7.69) | 6.85 (6.07－7.63) | 7.40 (6.50－8.30) |  | 2.71 (2.34－3.08) | 3.94 (3.33－4.54) | 1.20 (0.82－1.57) |
| Underweight | 2.48 (0.52－4.44) | 2.04 (0－4.33) | 3.16 (0－6.67) |  | 0.83 (0－1.97) | 0.68 (0－2.01) | 1.05 (0－3.10) |
| Normal weight | 3.79 (3.28－4.29) | 2.37 (1.81－2.93) | 5.28 (4.43－6.13) |  | 0.89 (0.64－1.14) | 1.03 (0.66－1.4) | 0.75 (0.42－1.08) |
| Overweight | 12.58 (10.61－14.55) | 10.63 (8.32－12.93) | 15.92 (12.34－19.50) |  | 4.96 (3.67－6.25) | 6.26 (4.45－8.07) | 2.74 (1.14－4.33) |
| Obesity | 36.91 (32.44－41.39) | 36.97 (31.97－41.98) | 36.67 (26.71－46.62) |  | 20.58 (16.83－24.33) | 23.81 (19.39－28.23) | 7.78 (2.24－13.31) |

The prevalence of elevated ALAT is expressed as % (95% confidence interval).

**Table S2.** The AUC (95% CI) of the ROC curve stratified by gender in univariate analyses, separately based on the diagnostic criteria I and II for elevated ALAT.

| Variable | Criterion I (>30U/L for boys and >19U/L for girls) | | | |  | Criterion II (>40U/L for boys and girls) | | | |
| --- | --- | --- | --- | --- | --- | --- | --- | --- | --- |
|  | AUC | 95% CI | SE | *P*-value^*^ |  | AUC | 95% CI | SE | *P*-value^*^ |
| Boys |  |  |  |  |  |  |  |  |  |
| Age | 0.631 | 0.599－0.663 | 0.016 | <0.001 |  | 0.634 | 0.594－0.675 | 0.021 | <0.001 |
| Height | 0.655 | 0.624－0.685 | 0.016 | <0.001 |  | 0.672 | 0.633－0.711 | 0.020 | <0.001 |
| **Weight** | **0.861** | **0.836－0.885** | **0.013** | **<0.001** |  | **0.878** | **0.849－0.907** | **0.015** | **<0.001** |
| **BMI** | **0.863** | **0.838－0.889** | **0.013** | **<0.001** |  | **0.884** | **0.856－0.913** | **0.015** | **<0.001** |
| Height-z | 0.497 | 0.463－0.531 | 0.017 | 0.863 |  | 0.504 | 0.461－0.548 | 0.022 | 0.852 |
| **BMI-z** | **0.825** | **0.796－0.854** | **0.015** | **<0.001** |  | **0.850** | **0.816－0.883** | **0.017** | **<0.001** |
| Girls |  |  |  |  |  |  |  |  |  |
| Age | 0.582 | 0.546－0.618 | 0.019 | <0.001 |  | 0.527 | 0.454－0.601 | 0.037 | 0.557 |
| Height | 0.595 | 0.554－0.636 | 0.021 | <0.001 |  | 0.551 | 0.455－0.647 | 0.049 | 0.271 |
| **Weight** | **0.716** | **0.678－0.754** | **0.019** | **<0.001** |  | **0.685** | **0.586－0.785** | **0.051** | **<0.001** |
| **BMI** | **0.705** | **0.667－0.743** | **0.019** | **<0.001** |  | **0.686** | **0.588－0.784** | **0.050** | **<0.001** |
| Height-z | 0.554 | 0.515－0.592 | 0.020 | 0.006 |  | 0.509 | 0.415－0.604 | 0.048 | 0.843 |
| BMI-z | 0.667 | 0.627－0.707 | 0.020 | <0.001 |  | 0.664 | 0.562－0.765 | 0.052 | <0.001 |

AUC, area under curve; ROC, receiver operating characteristic; ALAT, alanine aminotransferase; CI, confidence interval; SE, standard error.

^*^ Null hypothesis: true area under curve = 0.5.

**Table S3.** Spearman's rank correlation coefficients between individual measurements and ALAT.

| Variable | Age | Gender | Height | Weight | BMI | Height-z | BMI-z | ALAT |
| --- | --- | --- | --- | --- | --- | --- | --- | --- |
| Gender | 0.008 | 1 |  |  |  |  |  |  |
| Height | 0.398^**^ | 0.332^**^ | 1 |  |  |  |  |  |
| Weight | 0.345^**^ | 0.217^**^ | **0.673^**^** | 1 |  |  |  |  |
| BMI | 0.182^**^ | 0.045^**^ | 0.189^**^ | **0.829^**^** | 1 |  |  |  |
| Height-z | -0.469^**^ | 0.052 | 0.475^**^ | 0.249^**^ | -0.012 | 1 |  |  |
| BMI-z | -0.230^**^ | 0.101 | 0.028^*^ | **0.657^**^** | **0.891^**^** | 0.198^**^ | 1 |  |
| ALAT | 0.091^**^ | 0.220^**^ | 0.229^**^ | 0.359^**^ | 0.309^**^ | 0.065^**^ | 0.277^**^ | 1 |

^*^ 0.001 ≤ *P*-value < 0.05.

^**^ *P*-value < 0.001.
